# Supplementary material for: PKD2L1 channels segregated to the apical compartment are the dual-mode pH sensor in cerebrospinal fluid-contacting neurons
Source: eLife. 2026 Sep 29;15:RP109372. doi: 10.7554/eLife.109372 (PMC13623376; doi:10.7554/eLife.109372)
Supplement: Peer review history

# Editor report: eLife Assessment

**DOI:** 10.7554/eLife.109372.4.sa0

**Authors:** Jimena Berni

**Affiliations:** University of Sussex United Kingdom

**Keywords:** Compelling, Fundamental

This is a **fundamental** study on the sensory roles of cerebrospinal-fluid-contacting neurons (CSF-cNs) in mammals, revealing how the apical extension is used as an amplifier of chemical changes in the content of the CSF. Specifically, the authors show **compelling** evidence that PKD2L1 is predominantly a pH-sensing channel in CSF-cNs and link its apical localization to dual phasic and sustained responses underlying CSF chemosensation.

# Referee report: Reviewer #1 (Public review)

**DOI:** 10.7554/eLife.109372.4.sa1

[Editors' note: this version has been assessed by the Reviewing Editor without further input from the original reviewers. The authors have addressed the comments raised in the previous round of review.]

This study by Vitar et al. probes the molecular identity and functional specialization of pH-sensing channels in cerebrospinal fluid-contacting neurons (CSFcNs). Combining patch-clamp electrophysiology, laser-based local acidification, immunohistochemistry, and confocal imaging, the authors propose that PKD2L1 channels localized to the apical protrusion (ApPr) function as the predominant dual-mode pH sensor in these cells.

The work establishes a compelling spatial-physiological link between channel localization and chemosensory behavior. The integration of optical and electrical approaches is technically strong, and the separation of phasic and sustained response modes offers a useful conceptual advance for understanding how CSF composition is monitored.

# Referee report: Reviewer #2 (Public review)

**DOI:** 10.7554/eLife.109372.4.sa2

Summary:

Cerebrospinal fluid contacting neurons (CSF-cNs) are GABAergic cells surrounding the spinal cord central canal (CC). In mammals, their soma lies sub-ependymally, with a dendritic-like apical extension (AP) terminating as a bulb inside the CC.

How this anatomy-soma and AP in distinct extracellular environments-relates to their multimodal CSF-sensing function remains unclear.

The authors confirm in the GATA3:GFP mice where these cells are labeled that CSFcNs exhibit prominent spontaneous electrical activity mediated by PKD2L1 (TRPP2) channels, non-selective cation channels with ~200 pS conductance modulated by protons and mechanical forces.

They investigated PKD2L1 pH sensitivity and its effects on CSFcN excitability. They uncovered that PKD2L1 generates both phasic and tonic currents, bidirectionally modulated by pH with high sensitivity near physiological values.

Combining electrophysiology (intact and isolated AP recordings) with elegant laser-photolysis, they show functional PKD2L1 channels localize specifically to the apical extension (AP).

This spatial segregation, coupled with PKD2L1's biophysical properties (high conductance, pH sensitivity) and the AP's unique features (very high input resistance), renders CSFcN excitability highly sensitive to PKD2L1 modulation. Their findings reveal how the AP's properties are optimised for its sensory role.

Strengths:

This is a very convincing demonstration using elegant and challenging approaches (uncaging, outside out patch of the AP) together to form a complete understanding on how these sensory cells can detect so finely the changes of pH in the CSF.

# Author comment: Author response

**DOI:** 10.7554/eLife.109372.4.sa3

**Authors:** Federico F Trigo, Magdalena Vitar, Daniel Prieto, Stavros Malas, Raúl E Russo

**Affiliations:** Instituto de Investigaciones Biológicas Clemente Estable Avenida Italia 3318, Montevideo 11600 Uruguay; Instituto de Investigaciones Biológicas Clemente Estable Montevideo Uruguay; Universidad de la República Montevideo Uruguay; The Cyprus Institute Nicosia Cyprus; Instituto de Investigaciones Biológicas Clemente Estable Montevideo Uruguay

The following is the authors’ response to the previous reviews.

**Public Reviews:**

**Reviewer #1 (Public review):**

This study by Vitar et al. probes the molecular identity and functional specialization of pH-sensing channels in cerebrospinal fluid-contacting neurons (CSFcNs). Combining patch-clamp electrophysiology, laser-based local acidification, immunohistochemistry, and confocal imaging, the authors propose that PKD2L1 channels localized to the apical protrusion (ApPr) function as the predominant dual-mode pH sensor in these cells.

The work establishes a compelling spatial-physiological link between channel localization and chemosensory behavior. The integration of optical and electrical approaches is technically strong, and the separation of phasic and sustained response modes offers a useful conceptual advance for understanding how CSF composition is monitored.

Comments on revised version:

I thank the authors for their extensive revisions and detailed responses to the reviewers' comments. The manuscript has been substantially improved, and most of the major concerns raised in the initial review have been adequately addressed. In particular, the additional analyses of PKD2L1 channel activity, the incorporation of physiologically relevant pH conditions, the clarification of ASIC involvement, and the expanded Discussion have significantly strengthened the study.

Major scientific concerns largely addressed:

Quantification of PKD2L1 channel activity

The authors appropriately addressed my previous concerns regarding the use of Po as the sole measure of channel activity. The inclusion of additional parameters such as apparent Po, open time, nmax, holding current, and membrane charge provides a more robust assessment of PKD2L1 activity and substantially strengthens the conclusions.

Physiological relevance of pH modulation

The inclusion of experiments at pH 6.5 and the additional analyses of holding current and resting membrane potential are valuable additions. These experiments considerably improve the physiological relevance of the study.

ASIC contribution

The additional pharmacological experiments using ASIC blockers are helpful and support the conclusion that the photolysis-evoked response in the apical process is predominantly mediated by PKD2L1 channels.

Functional implications

The expanded Discussion regarding Ca2+-dependent signaling, neurosecretion, and the potential physiological roles of CSFcNs considerably improves the manuscript.

Remaining concerns:

Continued overstatement regarding "exclusive" localization and function:

Although the authors softened some statements in the revised manuscript, the term "exclusive" remains in several key locations, including the title.

For example:

"PKD2L1 channels segregated to the apical compartment are the exclusive dual-mode pH sensor..."

The data clearly demonstrate strong enrichment of functional PKD2L1 channels in the apical process. However, the available evidence does not fully justify the term "exclusive," particularly because:

- PKD2L1 immunoreactivity is still detectable outside the apical process.

- ASIC-mediated responses are present in CSFcNs.

- The authors themselves use more appropriate terminology such as "predominantly located" in the Discussion.

Therefore, I recommend replacing "exclusive" with more conservative terminology such as:

- predominant

- predominantly localized

- enriche

- functionally segregated

throughout the manuscript, including the title, Abstract, Introduction, Results, and Discussion.

We agree with the reviewer that the world “exclusive” is misleading and should be replaced. Following the reviewer’s suggestions, we have deleted the word “exclusive from the title, which now reads: “PKD2L1 channels segregated to the apical compartment are the functional dual-mode pH sensors in cerebrospinal fluid-contacting neurons.”

In addition, the word “exclusive” has been changed with more conservative terminology in other parts of the text: lines 80, 420, 466 and 551.

Use of the term "tonic current"

The manuscript continues to use the term "PKD2L1 tonic current."

While the dibucaine-sensitive holding current is clearly present, the precise mechanism generating this current remains uncertain. Indeed, the authors themselves acknowledge in the Discussion that:

- an alternative conducting state may exist, or

- unresolved brief channel openings may account for the current.

Therefore, the data support the existence of a sustained PKD2L1-associated current, but do not yet definitively establish a distinct tonic gating mode of the channel.

I therefore recommend replacing:

"tonic current" with a more neutral expression such as:

- sustained current

- PKD2L1-associated holding current

- sustained PKD2L1-mediated current throughout the manuscript.

Continued use of "off-current" and "off-response":

The revised manuscript has improved considerably in this regard. However, the terms "off-current" and "off-response" still remain in portions of the text and figure legends.

Because the manuscript itself demonstrates that the response reflects recovery from transient acidification rather than a separate OFF signaling mechanism, these terms remain potentially misleading.

I recommend replacing them with terminology such as:

- photolysis-evoked PKD2L1 current

- recovery current

- proton-removal-induced current

throughout the manuscript, including figure legends.

We apologize, as the word “tonic” and the terminology “off-current” should have completely disappeared after the first round of revisions. We have now replaced those all along the text. “Tonic” has been replaced by “sustained”.

“Off-current” or “off-response” have been replaced by appropriate terms in lines: 339, 342, 544, 545, 546, 549, 552, 555, 556, 560, 576, 580, 585, 588, 807 and 929. We have nevertheless conserved the term “off-current” in line 552 as we are referring to terminology used by other authors.

Minor editorial corrections

Figure 1Bd Please change: "po" to "Po" for consistency with standard channel physiology nomenclature.

Figure 1Ca Please add units (mV) to the voltage labels shown on the left side of the traces.

Figure 3E Please change: "Norm po" to "Norm Po".

Figure 4Fb Please replace: "sec" with "s" to conform with SI unit conventions.

Done.

The authors have addressed the majority of my previous concerns and the manuscript has been substantially improved. The remaining issues are primarily related to terminology and overinterpretation rather than experimental deficiencies.

**Reviewer #2 (Public review):**

Summary:

Cerebrospinal fluid contacting neurons (CSF-cNs) are GABAergic cells surrounding the spinal cord central canal (CC). In mammals, their soma lies sub-ependymally, with a dendritic-like apical extension (AP) terminating as a bulb inside the CC.

How this anatomy-soma and AP in distinct extracellular environments-relates to their multimodal CSF-sensing function remains unclear.

The authors confirm in the GATA3:GFP mice where these cells are labeled that CSFcNs exhibit prominent spontaneous electrical activity mediated by PKD2L1 (TRPP2) channels, non-selective cation channels with ~200 pS conductance modulated by protons and mechanical forces.

They investigated PKD2L1 pH sensitivity and its effects on CSFcN excitability. They uncovered that PKD2L1 generates both phasic and tonic currents, bidirectionally modulated by pH with high sensitivity near physiological values.

Combining electrophysiology (intact and isolated AP recordings) with elegant laser-photolysis, they show functional PKD2L1 channels localize specifically to the apical extension (AP).

This spatial segregation, coupled with PKD2L1's biophysical properties (high conductance, pH sensitivity) and the AP's unique features (very high input resistance), renders CSFcN excitability highly sensitive to PKD2L1 modulation. Their findings reveal how the AP's properties are optimised for its sensory role.

Strengths:

This is a very convincing demonstration using elegant and challenging approaches (uncaging, outside out patch of the AP) together to form a complete understanding on how these sensory cells can detect so finely the changes of pH in the CSF.

Weaknesses:

Not weaknesses, there are only minor requests to complete the beautiful study.

(1) The apical extension's response to removal of acidification is nicely illustrated in Figure 4C,G. There's something puzzling there: while the response to Glutamate is immediate, the channel responses to H+ is extremely delayed by 100ms - 2s, and even sometimes came in bursts separated by few hundreds of ms. H+ diffuse even faster than glutamate. Why is that?

I don't quite understand how the response is so delayed & how to explain the recurring bursts of channel opening in the figure panel ?

The kinetic of the response to proton uncaging is analyzed in Figure 4E, where the charge of the current traces is plotted against time. What this analysis shows is that the response lasts a few hundred ms (τ 250 ms) and then the PKD2L1 activity increase subsides to baseline. The peak of the response is at 100 ms (Figure 4G), but the increase in activity happens as soon as the uncaging pulse ends (Figure 4D, G and H). This behavior has already been shown in expression systems, where the channel activity is blocked by protons and the blockage is released when the acid is withdrawn. In an intact cell as the CSFcNs studied here, the exact kinetics of the recovery response are probably more complex (and variable) than in expression systems. Indeed, it is known that the recovery of this current depends, for example, on pH and extracellular calcium. Also, PKD2L1 are inhibited by intracellular calcium (de Caen et al, eLife 2016) but are themselves permeable to Ca^++^ ions. The interaction of these effects could give rise to the “bursts” that are observed in some cases. However, this is merely speculative at this point.

- The authors should show in Fig 4C,G the traces for 1-2 s before uncaging occurs so we can appreciate whether such events occur as well in baseline and discuss this further in revisions.

Following the reviewer’s suggestion, we have added a trace in Figure 4C (upper blue trace) showing the spontaneous activity of the cell, prior to uncaging, as it is already shown for another example in Figure 4D.

- Could the authors use a fluorescent pH sensor to monitor pH in the extracellular space and in the cell ?

This is an important point that was already addressed by the reviewing editors in the previous round of revisions. Indeed, we have attempted to perform pH calibrations in the setup using the pHsensitive dye pyranine (or HPTS: 8-Hydroxypyrene-1,3,6-trisulfonic acid). HPTS is a very useful tool for pH calibrations in the physiological range: its pKa value is close to 7.2 and it can be used as a ratiometric dye (its fluorescence is pH-independent at 405–410 nm and pH-dependent at 450 nm). Unfortunately, the calibration under the conditions of a real experiment is not possible because the photolysis in the slice occurs in a tiny volume (approximately 1 µm³ in a total bath volume of more than 1 ml). In these conditions, the 405 nm uncaging pulse bleaches the dye in the photolysis spot and any useful information is lost. In addition, our imaging system is not fast enough to follow the pH change. As discussed in the Materials and Methods section, subsection “Estimation of the pH drop induced by photolysis” (line 791), the fast protonation of bicarbonate indicates that the pH change induced by the photolysis recovers in the submillisecond range.

- Could the authors investigate whether in the apical extension, PKD2L1 channels are mainly at the outer membrane in the apical extension OR whether many channels are located in inner membranes ?

PKD2L1 channels are probably subject to a high rate of turnover, and they are certainly localized in the plasma membrane of the apical process as well as in the inner membranes. Although this is a very interesting point, we believe it is out of the scope of this work.

(2) Suppl Fig 4 is very cool and should be moved to main figure. The coupling of Soma and AP is very tight, yet there is a clear difference in targeting of channels that respond to cues in the CSF. In the context of an intact spinal cord, we can wonder how and when the contribution from ASIC in the some would be relevant to physiology. Can the authors think of experiments with an intact central canal to test the sensitivity and condition of recruitment of pH sensing in the soma (ASIC) versus the apical extension (PKD2L1)?

We have followed the suggestion of the reviewer and have made Supplementary Figure 4 a main figure.

The fact that the normal interphase between the spinal cord parenchyma and the cc is lost is already acknowledged in the discussion, lines 486 to 489. As the reviewer suggests, PKD2L1 and ASIC channels seem both to be important in the response of CSFcN to pH changes. However, both channels are activated in very different physiological contexts, as is discussed in the section “The involvement of ASIC channels”. Keeping the central canal intact in order to be as close as possible to physiological conditions, as suggested by the reviewer, would be ideal. However, as CSFcNs are in the middle of the cord, it would require the use of optical techniques that allow to penetrate deep into the tissue (e.g., 2-photon microscopy) that unfortunately are not available in our labs.

(3) The Reissner fiber is missing after slicing the spinal cord. From our observations in fish, the fiber being under tension triggers lots of activity in CSF-cNs (Bellegarda et al Elife 2023) that also relies on PKD2L1 (Bohm et al NC 2016; Sternberg et al NC 2019). Could the authors discuss the contribution of the Reissner fiber to the PKD2L1 mediated modulation of CSFcN excitability ? Could the authors conceive a way to slice along the anteroposterior axis (sagitally) the spinal cord to keep the Reissner fiber in the central canal when recording CSF-cN apical extension ?

- The authors should show in Fig 4C,G the traces for 1-2 s before uncaging occurs so we can appreciate whether such events occur as well in baseline and discuss this further in revisions.

As discussed in the previous point, the in vitro slice preparation has technical limitations that are mainly related to the alterations of the normal structure of the tissue. Although keeping the Reissner fiber intact in a sagittal slice seems possible, accessing the CSFcNs with electrophysiological methods would still be a challenge.

We have now added a sentence in the Discussion, lines 561 to 564, where we discuss that CSFcN excitability is modulated by the Reissner fiber and that it remains to be explored whether in rodents the gating of PKD2L1 channels is modulated by the Reissner fibre, as has been shown in zebrafish.
